# Supplementary material for: Combined Spectroscopic and Computational Study of Nitrobenzene Activation on Non-Noble Metals-Based Mono- and Bimetallic Catalysts
Source: Nanomaterials (Basel). 2021 Aug 10;11(8):2037. doi: 10.3390/nano11082037 (PMC8398056; doi:10.3390/nano11082037)
Supplement: Supplementary file 1 [file nanomaterials-11-02037-s001.zip › nanomaterials-1330933-supplementary.pdf]

Supplementary Materials

Combined Spectroscopic and Computational Study of Nitrobenzene Activation on Non-Noble Metals-Based Mono- and Bimetallic Catalysts

Reisel Millán, María Dolores Soriano, Cristina Cerdá Moreno, Mercedes Boronat * and Patricia Concepción *

Instituto de Tecnología Química (UPV-CSIC), Universitat Politècnica de València—Consejo Superior de Investigaciones Científicas, Avda. de los Naranjos s/n, 46022 Valencia, Spain; reimilca@itq.upv.es (R.M.); mdosorod@itq.upv.es (M.D.S.); cricermo@itq.upv.es (C.C.M.)

* Correspondence: boronat@itq.upv.es (M.B.); pconcepc@upvnet.upv.es (P.C.)

Characterization of monometallic samples

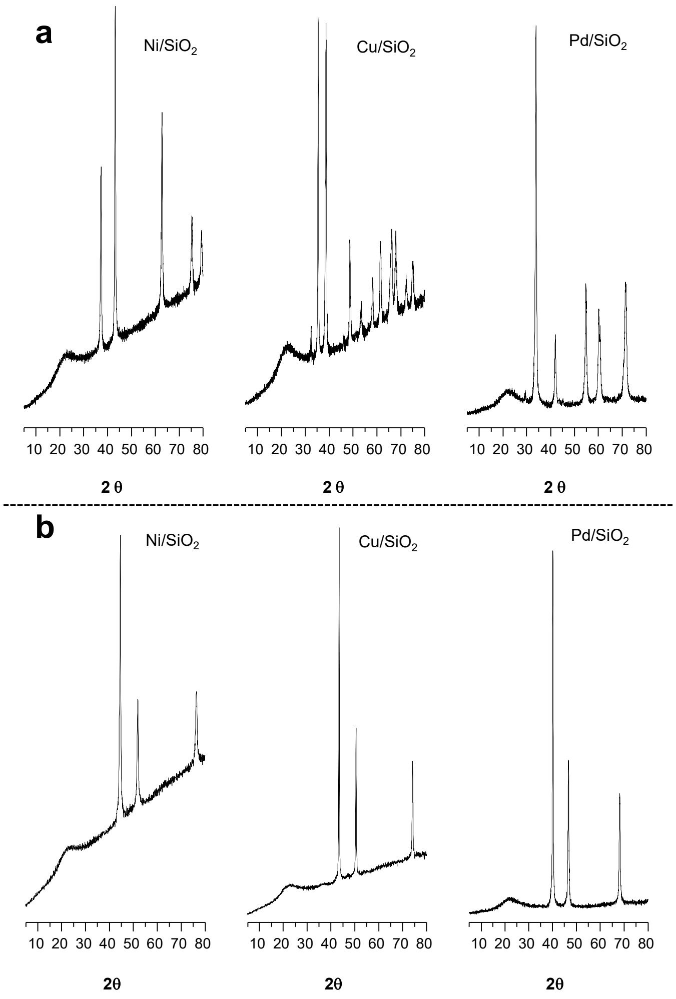

Figure S1. XRD pattern of the calcined (**a**) and (**b**) reduced monometallic Ni/SiO_2, Cu/SiO_2 and Pd/SiO_2 catalysts.

IR Study of nitrobenzene hydrogenation on monometallic samples

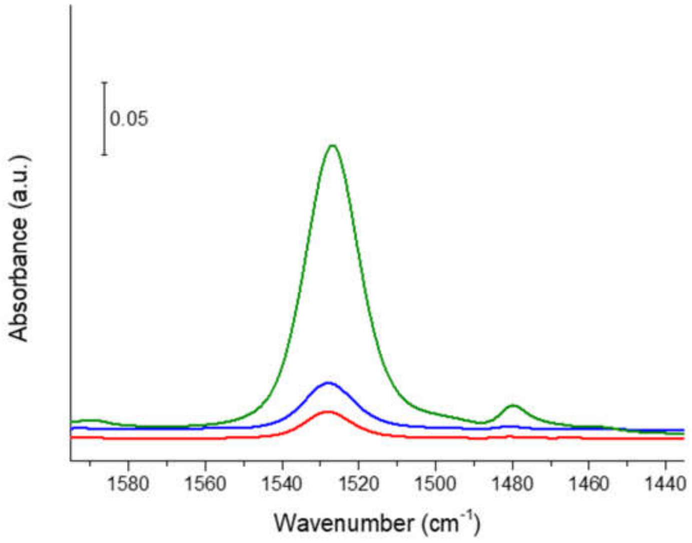

Figure S2. IR spectra of nitrobenzene adsorption at saturation coverage on Cu/SiO_2 (blue), Pd/SiO_2 (red), Ni/SiO_2 (green). Spectra normalized to sample weight. The lower intensity of the IR signal in the Cu/SiO_2 and Pd/SiO_2 samples agrees with the low interaction of nitrobenzene on both metals.

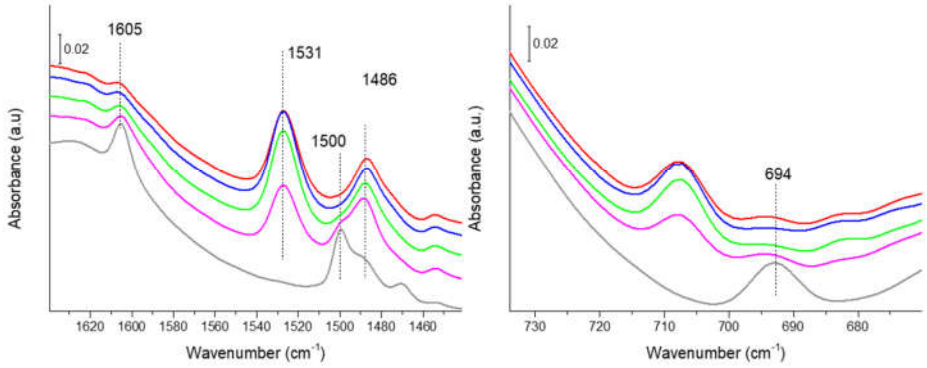

Figure S3. IR spectra of nitrosobenzene formed on the Ni/SiO_2 catalyst and after co-addition of H_2 at 25 °C (red line). Then, the temperature is increased to 90 °C (blue), 120 °C (green), 160 °C (magenta) and 200 °C (grey). Aniline formation (IR band at 1605, 1500 and 694 cm^{-1}) is detected at 200 °C.

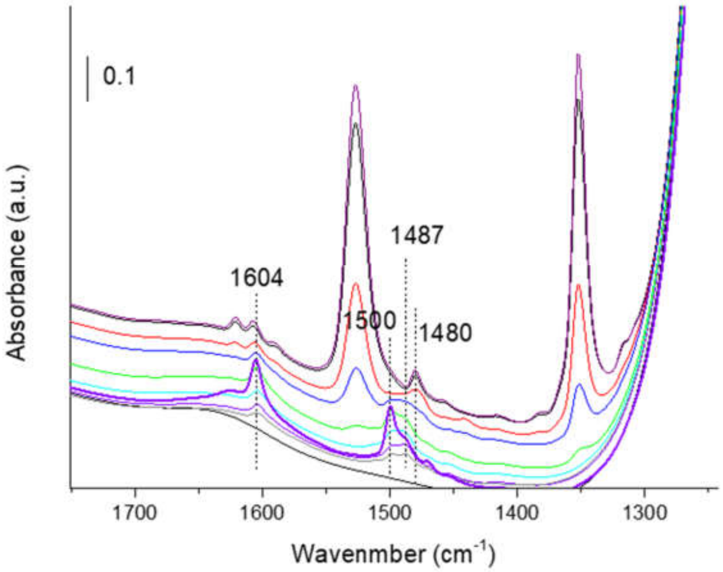

Figure S4. IR spectra of nitrobenzene and H_2 co-adsorbed on Ni/SiO_2, and their reactivity at increasing reaction temperature: 25 °C (black), 60 °C (red), 90 °C (blue), 120 °C (green), 140 °C (cyan), 180 °C (violet), 200 °C (grey). In violet bold after cooling down the sample from 180 ºC to 25 ºC allowing re-adsorption of the gas phase.

Characterization of bimetallic samples

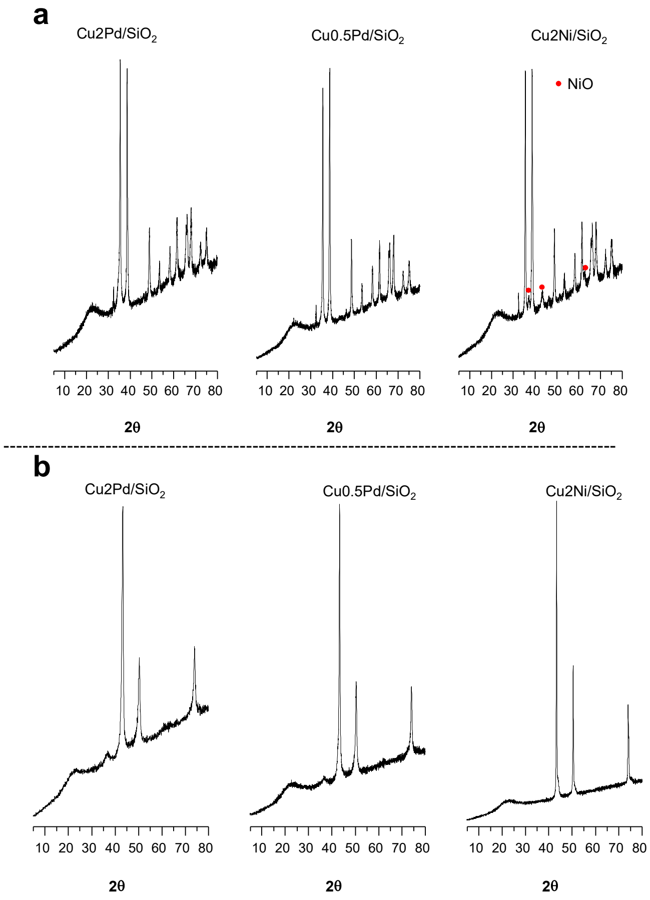

Figure S5. XRD pattern of the calcined (**a**) and (**b**) reduced bimetallic Cu2Pd/SiO_2, Cu0.5Pd/SiO_2 and Cu2Ni/SiO_2 catalysts.

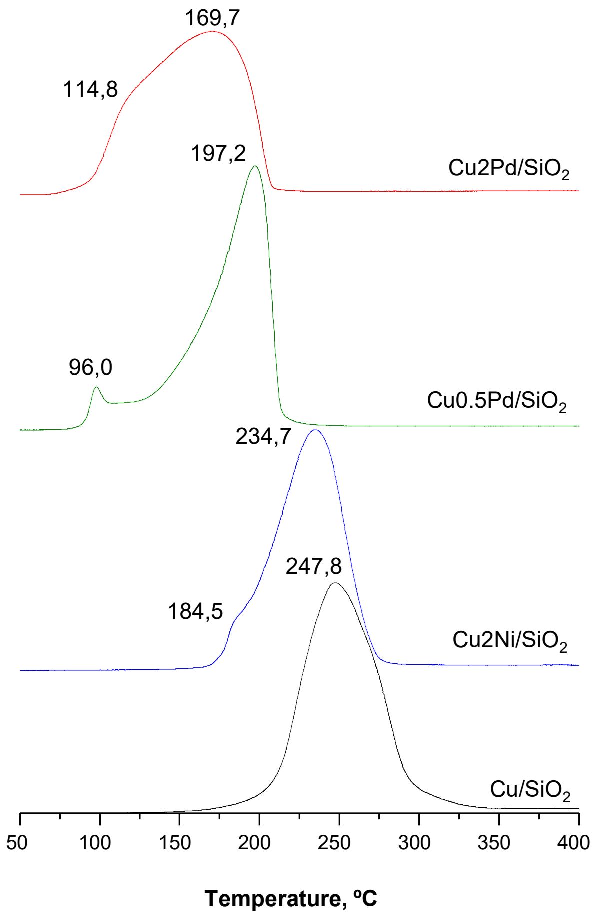

Figure S6. TPR-H_2 of monometallic Cu/SiO_2 and bimetallic Cu2Pd/SiO_2, Cu0.5Pd/SiO_2 and Cu2Ni/SiO_2 catalysts . The addition of Pd or Ni to the Cu monometallic catalyst, favour H_2 activation (as determined from the H_2D_2 isotopic exchange experiments), resulting in a higher catalyst reducibility.

Table S1. Surface composition determined by XPS.

sample	Si: Pd (Ni): Cu	Pd(Ni)/Cu
Cu0.5Pd/SiO_2	97: 0.5: 2.5	0.2
Cu2Pd/SiO_2	96: 0.7: 3.4	0.2
Cu2Ni/SiO_2	98: 0.6: 0.7	0.9

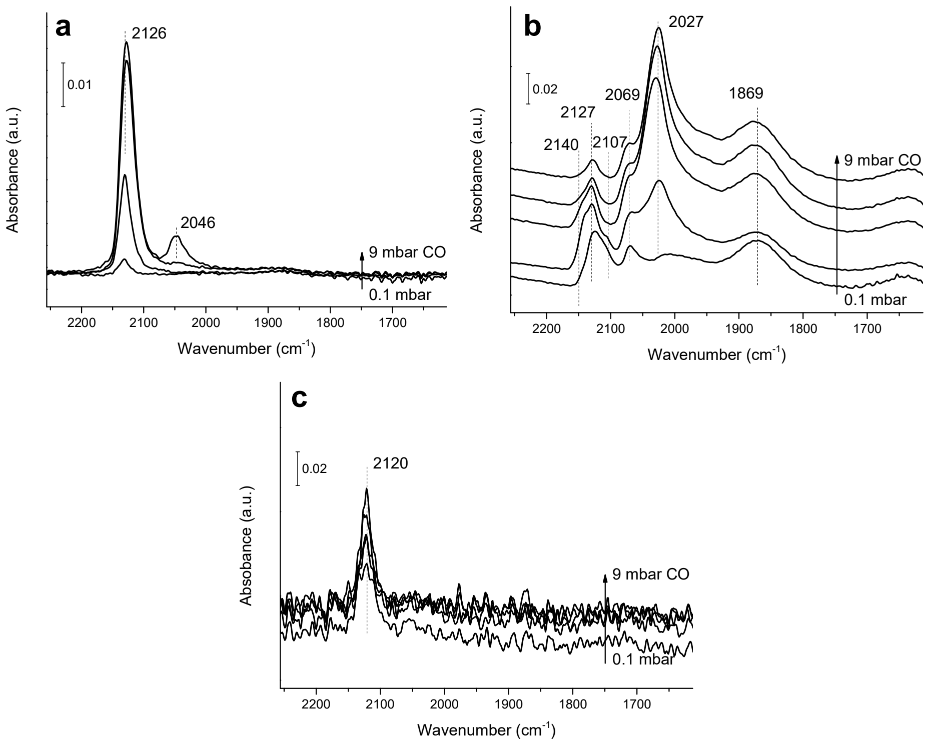

Figure S7. IR spectra of CO adsorption at -170 °C on (**a**) $Cu0.5Pd/SiO_2$, (**b**) $Cu2Pd/SiO_2$, (**c**) $Cu2Ni/SiO_2$.
